# Synthesis of Novel 2-(Substituted amino)alkylthiopyrimidin-4(3*H*)-ones as Potential Antimicrobial Agents

**DOI:** 10.3390/molecules19010279

**Published:** 2013-12-27

**Authors:** Mohamed I. Attia, Ali A. El-Emam, Abdulghafoor A. Al-Turkistani, Amany L. Kansoh, Nasser R. El-Brollosy

**Affiliations:** 1 Department of Pharmaceutical Chemistry, College of Pharmacy, King Saud University, P. O. Box 2457, Riyadh 11451, Saudi Arabia; 2 Microbial Chemistry Department, Genetic Engineering and Biotechnology Division, National Research Centre, Giza 12622, Egypt; 3 Department of Chemistry, Faculty of Science, Tanta University, Tanta 31527, Egypt

**Keywords:** 2-thiouracils, pyrimidin-4(3*H*)-ones, alkylation, antibacterial activity, anti-fungal activity

## Abstract

5-Alkyl-6-(substituted benzyl)-2-thiouracils **3a**,**c** were reacted with (2-chloroethyl) diethylamine hydrochloride to afford the corresponding 2-(2-diethylamino)ethylthiopyrimidin-4(3*H*)-ones **4a**,**b**. Reaction of **3a**–**c** with *N*-(2-chloroethyl)pyrrolidine hydrochloride and/or *N*-(2-chloroethyl)piperidine hydrochloride gave the corresponding 2-[2-(pyrrolidin-1-yl)ethyl]-thiopyrimidin-4(3*H*)-ones **5a**–**c** and 2-[2-(piperidin-1-yl)ethyl]thiopyrimidin-4(3*H*)-ones **6a**,**b**, respectively. Treatment of **3a**–**d** with *N*-(2-chloroethyl)morpholine hydrochloride under the same reaction conditions formed the corresponding 2-[2-(morpholin-4-yl)ethyl]thiopyrimidines **6c**–**f**. On the other hand, **3a**,**b** were reacted with *N*-(2-bromoethyl)phthalimide and/or *N*-(3-bromopropyl)phthalimide to furnish the corresponding 2-[2-(*N*-phthalimido)ethyl]-pyrimidines **7a**,**b** and 2-[3-(*N*-phthalimido)-propyl]pyrimidines **7c**,**d**, respectively. Compounds **3a**–**d**, **4a**,**b**, **5a**–**c**, **6a**–**f** and **7a**–**d** were screened against Gram-positive bacteria (*Staphylococcus aureus* ATCC 29213, *Bacillus subtilis* NRRL 4219 and *Bacillus cereus*), yeast-like pathogenic fungus (*Candida albicans* ATCC 10231) and a fungus (*Aspergillusniger* NRRL 599). The best antibacterial activity was displayed by compounds **3a**, **3b**, **4a**, **5a**, **5b**, **6d**, **6f**, **7b** and **7d**, whereas compounds **4b**, **5b**, **5c**, **6a**, **6b** and **6f** exhibited the best antifungal activity.

## 1. Introduction

In chemotherapy pyrimidines are considered as privileged structures with a large spectrum of biological activities. They are known very widely in Nature since they are components of RNA and DNA. The chemotherapeutic efficacy of pyrimidines may be due to their ability to inhibit vital enzymes responsible for nucleic acid biosynthesis such as reverse transcriptase, dihydrofolate reductase, uridine and thymidine phosphorylase, as well as thymidylate synthetase. Several pyrimidine derivatives exhibit diverse pharmacological activities as antiviral [1,2,3,4,5,6,7,8,9], anti-inflammatory [10,11,12], and antimalarial agents [13,14,15]. Many pyrimidines have been demonstrated to possess anticancer [16,17,18,19,20,21], antituberculosis [22] and anti-allergic [23] activities. Moreover, several pyrimidine derivatives have been reported as antithyroid [24] and antimicrobial agents [25,26,27,28,29,30], as well as human thymidine and uridine phosphorylase inhibitors [31,32,33].

In a previous study [34], we synthesized a series of 2-(substituted amino)ethylthiopyrimidines analogues of *S*-DABOs to be screened as reverse transcriptase inhibitors against human immunodeficiency virus (HIV-1). We found it of interest to evaluate the antimicrobial activity for such pyrimidine derivatives. In the present work, and as a part of our continuing interest in the chemistry of pyrimidines [30,34,35,36,37,38,39,40,41,42], the synthesis and antimicrobial evaluation of some novel 2-(substituted amino)alkylthiopyrimidin-4(3*H*)-one derivatives have been investigated.

## 2. Results and Discussion

### 2.1. Chemistry

**Scheme 1:**
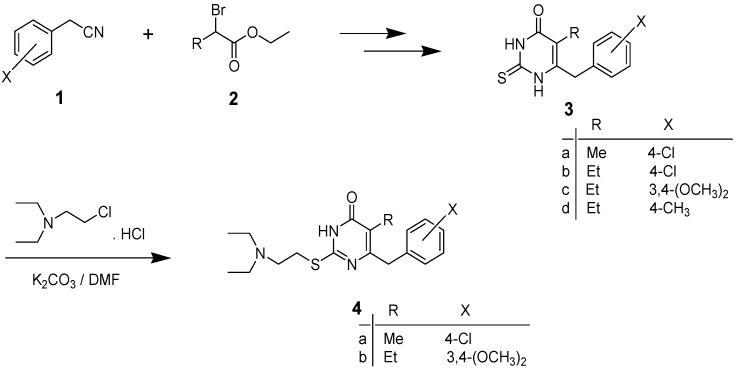
Synthesis of compounds **3a**–**d** and **4a**,**b**.

5-Alkyl-6-(substituted benzyl)-2-thiouracils **3a**–**d** were prepared, as described in our previous work [34,42], by reaction of (substituted phenyl)acetonitrile 1 with the appropriate ethyl 2-bromoesters **2** in anhydrous THF in the presence of zinc dust, followed by treatment of the β-ketoesters thus formed with thiourea in the presence of sodium ethoxide. Compounds **3a** and **3c** were reacted with (2-chloroethyl) diethylamine hydrochloride in DMF in the presence of anhydrous potassium carbonate to afford 6-(4-chlorobenzyl)-2-(2-diethylamino)ethylthio-5-methylpyrimidin-4(3*H*)-one (**4a**) [34] and 6-(3,4-dimethoxybenzyl)-2-(2-diethylamino)ethylthio-5-ethylpyrimidin-4(3*H*)-one (**4b**) in good yields (Scheme 1).

**Scheme 2:**
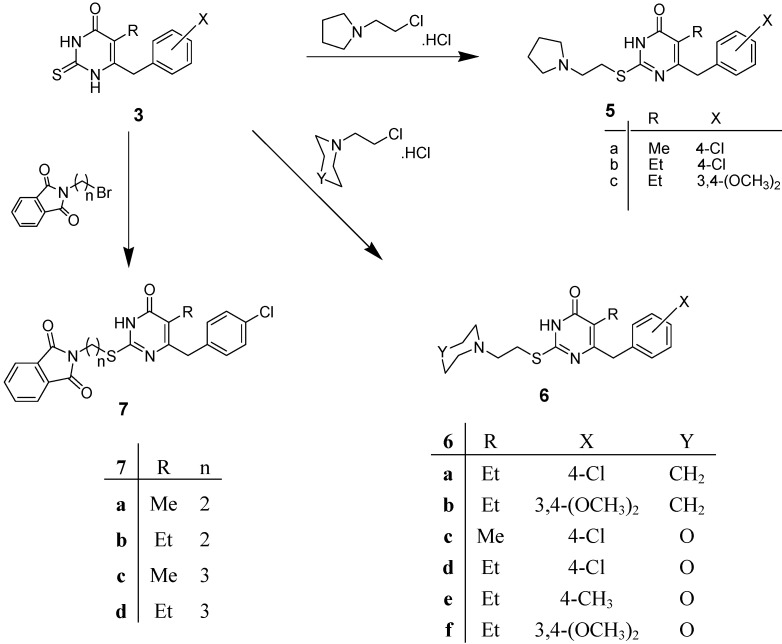
Synthesis of compounds **5a**–**c**, **6a**–**f** and **7a**–**d**.

6-(4-Chlorobenzyl)-5-methyl-2-[2-(pyrrolidin-1-yl)ethyl]thiopyrimidin-4(3*H*)-one (**5a**) [34], 6-(4-chlorobenzyl)-5-ethyl-2-[2-(pyrrolidin-1-yl)ethyl]thiopyrimidin-4(3*H*)-one (**5b**) [34] and 6-(3,4-dimethoxybenzyl)-5-ethyl-2-[2-(pyrrolidin-1-yl)ethyl]thiopyrimidin-4(3*H*)-one (**5c**) were obtained, respectively, in good yields, on reaction of compounds **3a**, **3b** and/or **3c** with *N*-(2-chloroethyl)pyrrolidine hydrochloride in the presence of anhydrous potassium carbonate in DMF (Scheme 2). Alkylation of **3b** and/or **3c** with *N*-(2-chloroethyl)piperidine hydrochloride in DMF containing potassium carbonate gave 6-(4-chlorobenzyl)-5-ethyl-2-[2-(piperidin-1-yl)ethyl]thiopyrimidin-4(3*H*)-one (**6a**) [34] and 6-(3,4-dimethoxybenzyl)-5-ethyl-2-[2-(piperidin-1-yl)ethyl]thiopyrimidin-4(3*H*)-one (**6b**) in 77% and 72% yields, respectively. Reaction of compounds **3a**–**d** with *N*-(2-chloroethyl)morpholine hydrochloride under the same reaction conditions formed the corresponding 2-[2-(morpholin-4-yl)ethyl]thiopyrimidines **6c**–**f** in 63%–74% yields (Scheme 2).

On the other hand, compounds **3a** and **3b** were treated with *N*-(2-bromoethyl)phthalimide and/or *N*-(3-bromopropyl)phthalimide in the presence of potassium carbonate in DMF to furnish the corresponding 2-[2-(*N*-phthalimido)ethyl]pyrimidines **7a**,**b** and 2-[3-(*N*-phthalimido)propyl]-pyrimidines **7c**,**d** in 69%, 71% and 62%, 64% yields, respectively (Scheme 2).

### 2.2. Antimicrobial Testing

The antimicrobial activities of the synthesized compounds, **3a**–**d**, **4a**,**b**, **5a**–**c**, **6a**–**f** and **7a**–**d** (200 µg/10 mm disc) as well as the reference drugs, ampicillin and clotrimazole, were screened against yeast-like pathogenic fungus (*Candida albicans* ATCC 10231), fungus (*Aspergillus niger* NRRL 599) and Gram-positive bacteria (*Staphylococcus aureus* ATCC 29213, *Bacillus subtilis* NRRL 4219 and *Bacillus cereus*) which are important human pathogenic microorganisms. A Diameter of Inhibition Zone (DIZ) assay [43] was performed to evaluate the preliminary antimicrobial potential of the test compounds against the test organisms and the results are given in Table 1.

**Table 1:** Antimicrobial activity of compounds **3a**–**d**, **4a**,**b**, **5a**–**c**, **6a**–**f** and **7a**–**d**, the broad spectrum antibacterial drug ampicillin and the antifungal drug clotrimazole against Gram-positive bacteria (*Staphylococcus aureus* ATCC 29213, *Bacillus subtilis* NRRL 4219 and *Bacillus cereus*), yeast-like pathogenic fungus (*Candida albicans* ATCC 10231) and fungus (*Aspergillus niger* NRRL 599).

Comp. No.	Diameter of Growth Inhibition Zone (mm) *^a^*				
	*Staphylococcus aureus*	*Bacillus subtilis*	*Bacillus cereus*	*Candida albicans*	*Aspergillus niger*
**3a**	22	17	21	15	-
**3b**	30	18	18	22	-
**3c**	-	-	-	-	-
**3d**	-	-	-	-	-
**4a**	30	12	12	-	13
**4b**	-	-	-	21	25
**5a**	30	15	13	13	-
**5b**	27	13	15	32	13
**5c**	-	-	-	35	31
**6a**	23	-	-	30	21
**6b**	23	-	-	20	25
**6c**	24	-	-	-	-
**6d**	27	18	12	15	-
**6e**	-	12	12	-	-
**6f**	21	15	13	25	24
**7a**	21	-	-	22	-
**7b**	25	13	12	12	-
**7c**	-	-	-	13	18
**7d**	24	13	16	12	-
**Ampicillin**	35	38	35		
**Clotrimazole**				38	40

*^a^* (-): Inactive (inhibition zone < 10 mm).

The synthesized compounds showed varying degrees of inhibition zones against the tested microorganisms. The antibacterial results revealed that compounds **3a**, **3b**, **4a**, **5a**, **5b**, **6a**–**d**, **6f**, **7a**, **7b** and **7d** showed strong activity (growth inhibition zones > 18 mm against one or more of the tested microorganisms), compound **6e** exhibited weak activity (growth inhibition zone 10–13 mm), while compounds **3c**, **3d**, **4b**, **5c** and **7c** showed no antibacterial activity (growth inhibition zones < 10 mm). Concerning the antifungal results, compounds **3b**, **4b**, **5b**, **5c**, **6a**, **6b**, **6f** and **7a** exhibited strong activity, compounds **3a**, **6d** and **7c** showed moderate activity (growth inhibition zones 14–18 mm), compounds **4a**, **5a**, **7b** and **7d** showed weak activity, whereas no antifungal activity was noticed for compounds **3c**, **3d**, **6c** and **6e**. In general, the best antibacterial activity was displayed by compounds **3a**, **3b**, **4a**, **5a**, **5b**, **6d**, **6f**, **7b** and **7d**. Compounds **4b**, **5b**, **5c**, **6a**, **6b** and **6f** exhibited the best antifungal activity, whereas compounds **3c** and **3d** showed no activity against the test organisms. Gram-positive bacteria, *Staphylococcus aureus*, and the yeast-like, *Candida albicans*, are considered the most sensitive among the tested microorganisms. The synthesized test compounds showed no activity against Gram negative pathogens, *Escherichia coli* and *Pseudomonas aeruginosa*. Although several compounds showed strong antibacterial and antifungal activities, none of them were found to be superior to the reference drugs. Compounds **3a**, **3b**, **4a**, **5a**, **5b**, **6b**, **6d**, **6f**, **7b** and **7d** displayed a relatively broad spectrum activity, accordingly, their MIC values were determined. The MIC values for compounds **3a**, **3b**, **4a**, **5a**, **5b**, **6b**, **6d**, **6f**, **7b** and **7d** against the most sensitive tested microorganisms, *Staphylococcus aureus* and *Candida albicans* are represented in Table 2.

**Table 2:** The minimal inhibitory concentration (MIC, µg/mL) values for compounds **3a**, **3b**, **4a**, **5a**, **5b**, **6b**, **6d**, **6f**, **7b** and **7d** against the most sensitive tested microorganisms, *Staphylococcus aureus* and *Candida albicans*.

Compound No.	The minimal inhibitory concentration (MIC µg/mL) *^a^*	
	*Staphylococcus aureus*	*Candida albicans*
**3a**	100	100
**3b**	25	25
**4a**	25	ND
**5a**	25	100
**5b**	25	25
**6b**	100	50
**6d**	25	100
**6f**	50	50
**7b**	50	100
**7d**	50	100
**Ampicillin**	6.0	
**Clotrimazole**		6.0

*^a^* The lowest concentration of the test compound that inhibits the growth of microorganism (μg/mL). ND: not determined.

According to the above results, the antimicrobial activity seemed to be dependent on the nature of substituents. Compounds containing a 4-chlorobenzyl substituent at C-6 of the pyrimidine ring showed the best antibacterial activity, whereas, the best antifungal results were given by compounds containing 2-(pyrrolidin-1-yl)ethylthio and 2-(piperidin-1-yl)ethylthio substituents at C-2 of the pyrimidine ring. Concerning compounds **7a**–**d**, the ethyl group at C-5 of the ring was found to improve the antimicrobial activity.

## 3. Experimental

### 3.1. General

Melting points (°C) were measured in open glass capillaries using a Branstead 9100 Electrothermal melting point apparatus and are uncorrected. NMR spectra were obtained on a Bruker AC 500 Ultra Shield NMR spectrometer (Fällanden, Switzerland) operating at 500.13 MHz for ^1^H and 125.76 MHz for ^13^C, the chemical shifts are expressed in δ (ppm) downfield from tetramethylsilane (TMS) as internal standard; coupling constants (*J*) are expressed in Hz and signals are expressed as s (singlet), d (doublet), t (triplet), q (quartet), or m (multiplet). Electrospray ionization mass spectra (ESI-MS) were recorded on an Agilent 6410 Triple Quad tandem mass spectrometer (Santa Clara, CA, USA) at 4.0 kV for the positive ions. The progress of reactions was monitored by TLC (DC-alufolio 60 F254) from Merck, and visualization with ultraviolet light (UV) at 365 and 254 nm. For column chromatography Merck silica gel (0.040–0.063 mm) was used. The tested microorganisms were obtained from MIRCIN Cairo, Faculty of Agriculture, Ain Shams University, Cairo, Egypt. Bacteria, fungi and yeast-like fungi were cultivated on agar media of nutrient, Czapek’sdox and malt–extract, respectively. The reference drugs ampicillin trihydrate (CAS 7177-48-2) and clotrimazole (CAS 23593-75-1) were obtained from Sigma-Aldrich Chemie GmbH (Taufkirchen, Germany). Compounds **3a**–**d**, **4a**, **5a**,**b** and **6a** were reported in our previous studies [34,42].

### 3.2. General Procedure for Preparation of 2-(Substituted amino)ethylthiopyrimidines **4b**, **5c** and **6b**–**f**

To a solution of the appropriate compound **3a**–**d** (1 mmol) in anhydrous DMF (5 mL), was added anhydrous potassium carbonate (0.304 g, 2.2 mmol) followed by the appropriate 2-chloroethyl substituted amine hydrochloride (1.1 mmol). The mixture was stirred at room temperature for 24 h, then was diluted with H_2_O (100 mL) and extracted with diethyl ether (3 × 50 mL). The combined organic extract was washed with H_2_O (3 × 50 mL), dried (MgSO_4_) and evaporated under reduced pressure. The residue was chromatographed on silica gel column with CHCl_3_ to afford the target compounds.

*6-(3,4-Dimethoxybenzyl)-2-[2-(diethylamino)ethy]lthio-5-ethylpyrimidin-4(3H)-one* (**4b**) White solid. M.p.: 119–120 °C, Yield: 0.287 g (71%). ^1^H-NMR (CDCl_3_): δ = 0.89 (t, 3H, *J* = 7.0 Hz, CH_3_), 1.00–1.04 (m, 6H, 2 × CH_3_), 2.41 (q, 2H, *J* = 7.0 Hz, CH_2_), 2.71–2.74 (m, 4H, 2 × CH_2_), 2.89–2.91 (m, 2H, CH_2_), 3.03–3.06 (m, 2H, CH_2_), 3.67 (s, 2H, CH_2_), 3.77 (s, 3H, OCH_3_), 3.78 (s, 3H, OCH_3_), 6.70 (bs, 2H, H_arom._), 6.77 (s, 1H, H_arom._), 11.71 (bs, 1H, NH). ^13^C-NMR (CDCl_3_): δ = 10.06 (CH_3_), 10.16 (CH_3_), 13.49 (CH_3_), 18.89 (CH_2_), 36.27 (CH_2_), 39.78 (CH_2_), 47.05 (2 × CH_2_), 54.62 (CH_2_), 55.88 (2 × OCH_3_), 120.68 (C-5), 111.09, 112.22, 119.14, 130.93, 147.63, 148.84 (C_arom._), 157.59 (C-6), 161.32 (C-4), 164.69 (C-2). ESI-MS, *m/z* (Rel. Int.): 406 (M + H^+^, 78).

*6-(3,4-Dimethoxybenzyl)-5-ethyl-2-[2-(pyrrolidin-1-yl)ethyl]thiopyrimidin-4(3H)-one* (**5c**) White solid. M.p.: 143–145 °C, Yield: 0.274 g (68%). ^1^H-NMR (DMSO-*d_6_*): δ = 0.92 (t, 3H, *J* = 7.5 Hz, CH_3_), 1.68–1.71 (m, 4H, 2 × CH_2_), 2.37 (q, 2H, *J* = 7.5 Hz, CH_2_), 2.52–2.55 (m, 4H, 2 × CH_2_), 2.68 (t, 2H, *J* = 6.0 Hz, CH_2_), 3.20 (t, 2H, *J* = 6.0 Hz, CH_2_), 3.70 (s, 3H, OCH_3_), 3.72 (s, 3H, OCH_3_), 3.84 (s, 2H, CH_2_), 6.72–6.88 (m, 3H. H_arom._). ^13^C-NMR (DMSO-*d_6_*): δ = 13.40 (CH_3_), 18.14 (CH_2_), 23.08 (CH_2_), 28.72 (CH_2_), 35.11 (CH_2_), 53.21 (CH_2_), 54.96 (CH_2_), 55.39 (OCH_3_), 55.48 (OCH_3_), 120.52 (C-5), 111.83, 112.89, 120.57, 131.06, 147.29, 148.50 (C_arom._), 159.97 (C-6), 161.20 (C-4), 164.01 (C-2). ESI-MS, *m/z* (Rel. Int.): 404 (M + H^+^, 90).

*6-(3,4-Dimethoxybenzyl)-5-ethyl-2-[2-(piperidin-1-yl)ethyl]thiopyrimidin-4(3H)-one* (**6b**) White solid. M.p.: 127–129 °C, Yield: 0.301 g (72%). ^1^H-NMR (CDCl_3_): δ = 0.88 (t, 3H, *J* = 7.5 Hz, CH_3_), 1.45–1.47 (m, 2H, CH_2_), 1.78–1.80 (m, 4H, 2 × CH_2_), 2.39 (q, 2H, *J* = 7.5 Hz, CH_2_), 2.50–2.52 (m, 4H, 2 × CH_2_), 2.73 (t, 2H, *J* = 5.0 Hz, CH_2_), 2.99 (t, 2H, *J* = 5.0 Hz, CH_2_), 3.75 (s, 2H, CH_2_), 3.77 (s, 3H, OCH_3_), 3.79 (s, 3H, OCH_3_), 6.70 (s, 2H, H_arom._), 6.79 (s, 1H, H_arom._), 11.73 (s, 1H, NH). ^13^C-NMR (CDCl_3_): δ = 13.23 (CH_3_), 18.91 (CH_2_), 24.01, 24.43, 55.44 (C_piperidin._), 36.21 (CH_2_), 39.80 (CH_2_), 55.90 (2 × OCH_3_), 61.57 (CH_2_), 122.64 (C-5), 111.09, 112.23, 120.71, 130.88, 147.33, 148.52 (C_arom._), 157.07 (C-6), 162.85 (C-4), 164.51 (C-2). ESI-MS, *m/z* (Rel. Int.): 418 (M + H^+^, 85).

*6-(4-Chlorobenzyl)-5-methyl-2-[2-(morpholin-4-yl)ethyl]thiopyrimidin-4(3H)-one* (**6c**) White solid. M.p.:158–159 °C, Yield: 0.280 g (74%).^1^H-NMR (DMSO-*d_6_*): δ = 1.95 (s, 3H, CH_3_), 2.33 (t, 4H, *J* = 4.5 Hz, 2 × CH_2_), 2.46 (t, 2H, *J* = 7.0 Hz, CH_2_), 3.13 (t, 2H, *J* = 7.0 Hz, CH_2_), 3.54 (t, 4H, *J* = 4.5 Hz, 2 × CH_2_), 3.84 (s, 2H, CH_2_), 7.24, 7.32 (2 × d, 4H, *J* = 8.5 Hz, H_arom._), 12.61 (s, 1H, NH). ^13^C-NMR (DMSO-*d_6_*): δ = 10.31 (CH_3_), 26.70 (CH_2_), 34.17 (CH_2_), 52.86, 65.97 (C_morpholin._), 57.35 (CH_2_); 115.19 (C-5), 128.13, 130.00, 130.58, 137.24 (C_arom._), 157.15 (C-6), 159.80 (C-4), 163.34 (C-2). ESI-MS, *m/z* (Rel. Int.): 380 (M + H^+^, 100).

*6-(4-Chlorobenzyl)-5-ethyl-2-[2-(morpholin-4-yl)ethyl]thiopyrimidin-4(3H)-one* (**6d**) White solid. M.p.: 133–135 °C. Yield: 0.271 g (69%). ^1^H-NMR (DMSO-*d_6_*): δ = 0.94 (t, 3H, *J* = 7.5 Hz, CH_3_), 2.31 (t, 4H, *J* = 4.5 Hz, 2 × CH_2_), 2.43–2.46 (m, 4H, 2 × CH_2_), 3.12 (t, 2H, *J* = 7.0 Hz, CH_2_), 3.53 (t, 4H, *J* = 4.5 Hz, CH_2_), 3.85 (s, 2H, CH_2_), 7.25, 7.33 (2 × d, 4H, *J =* 8.5 Hz, H_arom._), 12.79 (s, 1H, NH).^13^C-NMR (DMSO-*d_6_*): δ = 13.16 (CH_3_), 18.02 (CH_2_), 26.64 (CH_2_), 30.59 (CH_2_), 52.82, 65.96 (C_morpholin._), 57.32 (CH_2_), 121.27 (C-5), 128.09, 130.64, 130.78, 137.55 (C_arom._), 157.20 (C-6), 159.44 (C-4), 162.77 (C-4). ESI-MS, *m/z* (Rel. Int.): 394 (M + H^+^, 100).

*5-Ethyl-6-(4-methylbenzyl)-2-[2-(morpholin-4-yl)ethyl]thiopyrimidin-4(3H)-one* (**6e**) White solid. M.p.: 137–139 °C.Yield: 0.236 g (63%). ^1^H-NMR (DMSO-*d_6_*): δ = 0.90 (t, 3H, *J* = 7.5 Hz, CH_3_), 2.25 (s, 3H, CH_3_), 2.31–2.39 (m, 6H, 3 × CH_2_), 2.58 (t, 2H, *J* = 7.0 Hz, CH_2_), 3.15 (t, 2H, *J* = 7.0 Hz, CH_2_), 3.51 (t, 4H, *J* = 4.5 Hz, 2 × CH_2_), 3.79 (s, 2H, CH_2_), 7.07, 7.11 (2 × d, 4H, *J* = 8.0 Hz, H_arom._), 12.49 (s, 1H, NH). ^13^C-NMR (DMSO-*d_6_*): δ = 13.14 (CH_3_), 18.08 (CH_2_), 20.51 (CH_3_), 26.62 (CH_2_), 52.83, 65.98 (C_morpholin._), 57.38 (CH_2_), 120.98 (C-5), 128.56, 128.76, 135.04, 135.41 (C_arom._), 157.93 (C-6), 162.20 (C-4), 164.63 (C-2). ESI-MS, *m/z* (Rel. Int.): 374 (M + H^+^, 100).

*6-(3,4-Dimethoxybenzyl)-5-ethyl-2-[2-(morpholin-4-yl)ethyl]thiopyrimidin-4(3H)-one* (**6f**) White solid. M.p.: 151–152 °C, Yield: 0.276 g (66%). ^1^H-NMR (DMSO-*d_6_*): δ = 0.90 (t, 3H, *J* = 7.5 Hz, CH_3_), 2.30–2.39 (m, 6H, 3 × CH_2_), 2.58 (t, 2H, *J* = 7.0 Hz, CH_2_), 3.21 (t, 2H, *J* = 7.0 Hz, CH_2_), 3.57 (t, 4H, *J* = 4.5 Hz, 2 × CH_2_), 3.70 (s, 3H, OCH_3_), 3.72 (s, 3H, OCH_3_), 3.76 (s, 2H, CH_2_), 6.72–6.87 (m, 3H, H_arom._), 12.54 (s, 1H, NH). ^13^C-NMR (DMSO-*d_6_*): δ = 13.32 (CH_3_), 18.06 (CH_2_), 26.68 (CH_2_), 52.83, 65.98 (C_morpholin._), 55.36 (OCH_3_), 55.42 (OCH_3_), 57.44 (CH_2_), 117.23 (C-5), 111.76, 112.85, 120.57, 131.29, 147.27, 148.45 (C_arom._), 158.34 (C-6), 162.20 (C-4), 162.43 (C-2). ESI-MS, *m/z* (Rel. Int.): 420 (M + H^+^, 100).

### 3.3. General Procedure for Preparation of 2-[2-(N-Phthalimido)ethyl]thiopyrimidin-4(3H)-ones **7a**,**b** and 2-[3-(N-Phthalimido)propyl]thiopyrimidin-4(3H)-ones **7c**,**d**

Anhydrous potassium carbonate (0.152 g, 1.1 mmol) was added to a solution of the appropriate compound **3a**,**b** (1 mmol) in DMF (5 mL), followed by addition of *N*-(2-bromoethyl)phthalimide and/or *N*-(3-bromopropyl)phthalimide (1.1 mmol). The reaction mixture was stirred at room temperature for 24 h and worked up as described above for the preparation of compounds **4**–**6**.

*6-(4-Chlorobenzyl)-5-methyl-2-[2-(N-phthalimido)ethyl]thiopyrimidin-4(3H)-one* (**7a**) White solid. M.p.: 251–253 °C, Yield: 0.302 g (69%). ^1^H-NMR (DMSO-*d_6_*): δ = 1.90 (s, 3H, CH_3_), 3.34 (t, 2H, *J* = 6.0 Hz, CH_2_), 3.82 (s, 2H, CH_2_), 3.87 (t, 2H, *J* = 6.0 Hz, CH_2_), 7.32–7.33 (m, 4H, H_arom._), 7.83–7.88 (m, 4H, H_arom._), 12.69 (s, 1H, NH). ^13^C-NMR (DMSO-*d_6_*): δ = 10.27 (CH_3_), 28.12 (CH_2_), 30.59 (CH_2_), 36.61 (CH_2_), 115.11 (C-5), 123.00, 128.15, 130.64, 130.83, 131.41, 134.36, 137.18 (C_arom._), 159.13 (C-6), 162.21 (C-4), 163.11 (C-2), 167.60 (CO). ESI-MS, *m/z* (Rel. Int.): 440 (M + H^+^, 18).

*6-(4-Chlorobenzyl)-5-ethyl-2-[2-(N-phthalimido)ethyl]thiopyrimidin-4(3H)-one* (**7b**) White solid. M.p.: 203–204 °C, Yield: 0.322 g (71%). ^1^H-NMR (DMSO-*d_6_*): δ = 0.87 (t, 3H, *J* = 7.5 Hz, CH_3_), 2.37 (q, 2H, *J* = 7.5 Hz, CH_2_), 3.35 (t, 2H, *J* = 6.0 Hz, CH_2_), 3.80 (s, 2H, CH_2_), 3.86 (t, 2H, *J* = 6.0 Hz, CH_2_), 7.33–7.34 (m, 4H, H_arom._), 7.83–7.89 (m, 4H, H_arom._), 12.71 (s, 1H, NH). ^13^C-NMR (DMSO-*d_6_*): δ = 13.03 (CH_3_), 18.00 (CH_2_), 28.07 (CH_2_), 30.60 (CH_2_), 36.71 (CH_2_), 115.19 (C-5), 123.00, 128.12, 130.70, 130.83, 131.40, 134.36, 137.50 (C_arom._), 162.34 (C-4), 163.22 (C-2), 167.60 (CO). ESI-MS, *m/z* (Rel. Int.): 454 (M + H^+^, 31).

*6-(4-Chlorobenzyl)-5-methyl-2-[3-(N-phthalimido)propyl]thiopyrimidin-4(3H)-one* (**7c**) White solid. M.p.: 234–235 °C, Yield: 0.282 g (62%). ^1^H-NMR (DMSO-*d_6_*): δ = 1.85–1.91 (m, 2H, CH_2_), 1.93 (s, 3H, CH_3_), 3.02 (t, 2H, *J* = 6.5 Hz, CH_2_), 3.60 (t, 2H, *J* = 6.5 Hz, CH_2_), 3.74 (s, 2H, CH_2_), 7.19, 7.25 (2 × d, 4H, *J* = 8.0 Hz, H_arom._), 7.80–7.85 (m, 4H, H_arom._), 12.72 (s, 1H, NH). ^13^C-NMR (DMSO-*d_6_*): δ = 10.29 (CH_3_), 26.97 (CH_2_), 28.13 (CH_2_), 30.59 (CH_2_), 36.37 (CH_2_), 115.32 (C-5), 122.88, 128.03, 130.53, 130.74, 131.55, 134.22, 137.18 (C_arom._), 157.86 (C-6), 162.71 (C-4), 163.67 (C-2), 167.87 (CO). ESI-MS, *m/z* (Rel. Int.): 454 (M + H^+^, 20).

*6-(4-Chlorobenzyl)-5-ethyl-2-[3-(N-phthalimido)propyl]thiopyrimidin-4(3H)-one* (**7d**) White solid, M.p.: 197–198 °C, Yield: 0.298 g (64%). ^1^H-NMR (DMSO-*d_6_*): δ = 0.93 (t, 3H, *J* = 7.5 Hz, CH_3_), 1.84–1.89 (m, 2H, CH_2_), 2.41 (q, 2H, *J* = 7.5 Hz, CH_2_), 3.02 (t, 2H, *J* = 6.5 Hz, CH_2_), 3.59 (t, 2H, *J* = 6.5 Hz, CH_2_), 3.76 (s, 2H, CH_2_), 7.21, 7.26 (2 x d, 4H, *J* = 8.0 Hz, H_arom._), 7.80–7.85 (m, 4H, H_arom._), 12.74 (s, 1H, NH). ^13^C-NMR (DMSO-*d_6_*): δ = 13.11 (CH_3_), 18.01 (CH_2_), 26.97 (CH_2_), 28.09 (CH_2_), 30.59 (CH_2_), 36.34 (CH_2_), 115.69 (C-5), 122.88, 128.00, 130.60, 130.74, 131.55, 134.21, 137.47 (C_arom._), 158.98 (C-6), 161.92 (C-2), 163.43 (C-4), 167.87 (CO). ESI-MS, *m/z* (Rel. Int.): 468 (M + H^+^, 17).

### 3.4. Determination of the Antimicrobial Activity by the Agar Disc-Diffusion Method [43]

Sterile nutrient, Czapek’s dox and malt extract agar media were inoculated, separately, with 100 µL cell suspension of the chosen microorganism, bacteria, fungi and yeast-like fungi, respectively, and poured into Petri-dishes (20 cm diameter). The test compounds (200 µg/10 mm diameter disc) were placed onto the surface of the agar Petri-dishes. The antimicrobial activities were expressed as the diameter of the growth inhibition zone in mm.

### 3.5. Determination of Minimal Inhibitory Concentration (MIC) [44]

The minimal inhibitory concentrations (MICs) of the test compounds were determined using serial dilutions technique. Different concentrations ranging 50.0–200.0 µg/mL for each compound in dimethyl sulphoxide (DMSO) were placed on filter paper disc (1 cm diameter). The discs were deposited on the surface of inoculated agar plates and kept at low temperature before incubation which favours diffusion over microbial growth to detect the inhibition zone clearly. The plates were incubated at 30 °C for 24 h for bacteria and yeast and for 48 h for fungi.

## 4. Conclusions

In the present study, several 2-(substituted amino)alkylthiopyrimidin-4(3*H*)-ones were synthesized and screened against Gram-positive bacteria (*Staphylococcus aureus* ATCC 29213, *Bacillus subtilis* NRRL 4219 and *Bacillus cereus*), yeast-like pathogenic fungus (*Candida albicans* ATCC 10231) and fungus (*Aspergillus niger* NRRL 599) which are important human pathogenic microorganisms. Most of the test compounds showed good antimicrobial activities.
